# Ethogram Characteristics of Silver Carp (*Hypophthalmichthys molitrix*) During the Breeding Period Based on the PAE Coding System

**DOI:** 10.3390/ani15091218

**Published:** 2025-04-25

**Authors:** Min Wang, Fengyue Zhu, Lixiong Yu, Qingrui Yang, Ke Wang, Mingdian Liu, Xinbin Duan, Daqing Chen

**Affiliations:** 1 Key Laboratory of Freshwater Fish Reproduction and Development (Ministry of Education), School of Life Science, Southwest University, Chongqing 400715, China; hznwangmin@163.com; 2 National Agricultural Science Observing and Experimental Station of Chongqing, Yangtze River Fisheries Research Institute, Chinese Academy of Fishery Science, Wuhan 430223, China; zhufy@yfi.ac.cn (F.Z.); yulixiong@yfi.ac.cn (L.Y.); wangke@yfi.ac.cn (K.W.); lmd@yfi.ac.cn (M.L.); duan@yfi.ac.cn (X.D.); 3 Department of Water Environment, China Institute of Water Resources and Hydropower Research, Beijing 100038, China; lzyqr2004@163.com

**Keywords:** *Hypophthalmichthys molitrix*, breeding period, PAE coding system, ethogram

## Abstract

Animal behavior diversity is a key element of biodiversity, and the establishment of an ethogram provides a crucial framework for exploring the relationship between behavioral patterns and environmental factors. However, the development of ethograms and the quantitative analysis of behavioral diversity in fish remain underexplored. In this study, focal animal sampling and instantaneous scan sampling methods, paired with the PAE (Posture-Act-Environment) coding system, were employed to investigate the ethogram and reproductive behavior diversity of silver carp. The findings revealed 12 postures, 20 actions, and 34 behaviors documented during the breeding period of silver carp. Analysis of reproductive behavior diversity revealed that the absolute behavioral diversity index (*H*), relative behavioral diversity index (*r*), and regulated diversity index (*r-variable*) all peaked during the 10–15 h interval following induced spawning. Notably, significant differences (*p* < 0.001) were observed in these indices across different post-induction time intervals and between sexes. Throughout the breeding cycle, females demonstrated lower diversity and intensity in reproductive behaviors compared to males, suggesting distinct reproductive strategies between the sexes. The reproductive behavior of silver carp exhibited distinct chronobiological disorganization patterns. This study provides a scientific basis for future research on the behavioral ecology of silver carp and the conservation of their wild populations.

## Simple Summary

The establishment of an ethogram for fish facilitates precise behavioral description and classification, contributing to the understanding of their functional roles and interrelationships. This study develops an ethogram and a PAE (Posture-Act-Environment) coding system for silver carp during the breeding season, examining the variation in reproductive behaviors at different time points post-induced spawning and between sexes. A total of 34 distinct behaviors were identified and categorized into five groups. Notable differences in behavioral patterns were observed across post-induction stages and between sexes, with males exhibiting higher activity levels and greater behavioral diversity throughout the breeding period. Behavioral diversity indices initially rose, then declined over time post-spawning, with significant sex-based differences. These results provide essential insights for constructing quantitative ethograms and advancing behavioral ecology research in other fish species.

## 1. Introduction

Animal behavior refers to a series of actions exhibited by animals in various postures within both biotic and abiotic environments, aimed at fulfilling survival and physiological needs [1]. It represents a coordinated response to internal physiological changes and external environmental stimuli. Investigating animal behavior uncovers activity patterns and is essential for understanding the environmental adaptation mechanisms that underpin different behavioral strategies [2,3]. An ethogram, defined as a catalog of behaviors, is created through the systematic observation, recording, identification, and classification of animal behaviors [4]. The development of an ethogram is a foundational aspect of quantitative animal behavior research, enabling a deeper exploration of the relationship between behavior and ecological functions [5,6].

Traditional ethogram research has predominantly relied on direct observation and descriptive definitions as its core methodologies [7,8]. These approaches have enabled the collection of comprehensive behavioral sequence datasets and laid the groundwork for animal behavior studies. However, as research subjects become more diverse—particularly in the study of aquatic animals—and as research demands increase, traditional methods show growing limitations in terms of observational precision, data standardization, and experimental reproducibility. In response, the PAE (Posture-Act-Environment) coding system emerged as an innovative behavioral analysis methodology. Developed in the 1990s by Jiang, the PAE coding classification system was introduced through significant work on behavioral patterns in Père David’s Deer (*Elaphurus davidianus*) [9]. This system categorizes animal behavior into three primary elements—postures, actions, and the ecological environment—and applies mathematical methods to encode these components. By standardizing behavioral data, the PAE system offers a structured framework for analyzing the hierarchical structure of animal behavior. Its highly structured nature makes the PAE system particularly advantageous for analyzing animal behavior in complex ecological contexts, providing a significant methodological innovation for research in aquatic animal ethology.

Since the introduction of the PAE coding classification system, its application has expanded to analyze the behavioral spectra of various terrestrial species, including the toad-headed lizard (*Phrynocephalus vlangalii*) [10], painted snipe (*Rostratula benghalensis*) [11], giant panda (*Ailuropoda melanoleuca*) [12], and Chinese pangolin (*Manis pentadactyla*) [13]. However, studies on the classification and coding of behaviors in aquatic animals remain limited. To date, ethograms based on the PAE system have been compiled for the Yangtze finless porpoise (*Neophocaena phocaenoides asiaeorientalis*) [14], *Schizothorax wangchiachii* [15], and *Odontobutis potamophila* [16]. Furthermore, research indicates that environmental factors, such as water temperature, flow velocity, and photoperiod, significantly affect the behavioral patterns of aquatic organisms, including fish [17,18]. Consequently, there is an increasing need to focus on the dynamic behavioral changes in aquatic animals.

The silver carp (*Hypophthalmichthys molitrix*) is an ecologically and commercially important species widely distributed across China’s river systems, with the middle reaches of the Yangtze River serving as a crucial habitat and breeding ground [19]. However, the construction and operation of water conservancy projects on the Yangtze River have disrupted the natural hydrological regime and environmental conditions downstream of the dams, severely impacting the habitats and reproductive activities of aquatic organisms. This has led to a significant decline in the spawning volumes of the four major Chinese carps (FMCCs), including the silver carp [20,21]. As such, comprehensive research on the conservation of silver carp populations throughout their life cycle is essential. Reproduction, a key element of fish life history, is intrinsically linked to population sustainability, and reproductive behavior plays a vital role in ensuring successful reproductive outcomes. However, prior studies on silver carp life history have primarily focused on early development [22], growth characteristics [23,24], feeding ecology [25], and population dynamics [26], with limited attention given to their reproductive behavioral patterns. Furthermore, despite being a keystone species in freshwater ecosystems, the silver carp lacks a standardized ethogram, particularly one based on the PAE framework, resulting in limited comparability of behavioral observation data across studies. To address this gap, this study simulated the natural breeding activities of silver carp in a large indoor circular flume and, for the first time, developed a behavioral ethogram of silver carp during the breeding period based on the PAE coding system. Additionally, a preliminary comparative analysis of reproductive behavior diversity was conducted among groups with different post-induction time intervals and between sexes. This research provides a scientific foundation for the behavioral ecology and conservation of wild silver carp populations while establishing a quantifiable and generalizable methodological framework for ethogram studies in freshwater fish species.

## 2. Materials and Methods

### 2.1. The Experimental Fish

A total of 49 sexually mature silver carp, consisting of 21 females and 28 males, were sourced from the national original breeding farm of the FMCCs in Jianli City, Hubei Province, China. The broodstock were transported from the breeding base to the laboratory using fish transport trucks equipped with an oxygenation system. Prior to the formal experiment, the silver carp were temporarily housed in several acclimation tanks (4.0 m × 2.0 m × 1.0 m) and fed phytoplankton-based feed once daily in the morning. The aquaculture system employed recirculating water sourced from the Laojiang River in Jianli (a former course of the Yangtze River), maintaining dissolved oxygen levels above 7 mg/L. Only disease-free fish exhibiting clear secondary sexual characteristics were selected for the breeding experiment. Males exhibited keratinized tubercles on the epidermal layer of the first two fin rays of the pectoral fins, which felt rough and spiny to the touch. Females had smooth pectoral fins, a distended and soft abdomen, and a slightly reddened genital papilla [27].

### 2.2. Laboratory Apparatus

**Figure 1:**
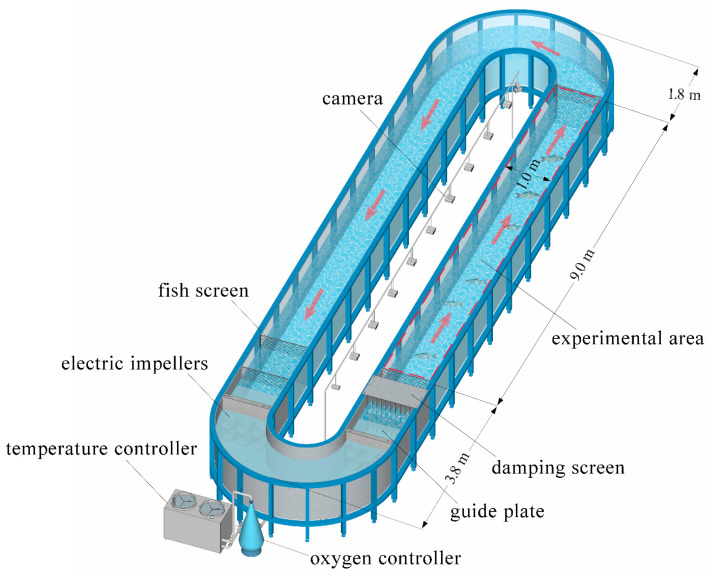
Schematic diagram of the reproductive experiment flume installation. This setup includes auxiliary facilities such as a temperature controller, an oxygen regulator, and a comprehensive monitoring system. The experimental section consists of a simulated fishway, measuring 9.0 m in length, 1.0 m in width, and 0.8 m in depth.

The experiment was conducted in a large annular flume (Figure 1) measuring 14.6 m in length, 3.6 m in width, and 1.5 m in height. The tank’s cross-sectional dimensions were 1 m × 1 m (width × depth), with an experimental water depth set at 0.8 m. The flume’s main structure included a stainless steel frame, a tempered glass tank body (fishway), a flow generation system, a temperature control system, and an oxygen supply system (CC-SC-YQZ60, Guangzhou Lanling Aquatic Technology Co., Ltd., Guangzhou, China). Electronically controlled impellers (180 lbf, 2700 W, Ningbo Youdong Electric Motor Co., Ltd., Ningbo, China) were installed along the curved section on one side of the flume, with six impellers on each side, primarily to regulate water flow velocity. Guide plates at both the front and rear ends of the impellers prevented air intake and water overflow, thereby enhancing operational efficiency. Damping screens were used to stabilize the turbulent water flow discharged by the impellers, ensuring a consistent water velocity within the fishway. The water temperature controller (KH430S YIE/A, Guangdong Tongyi Heat Pump Science and Technology CO., Ltd., Guangzhou, China) maintained the water temperature within an optimal range for silver carp reproduction. Fish screens were placed at the water inlet and outlet of the flume to prevent the fish from entering the impellers and being injured. Additionally, two fish screens were positioned along the straight section of the water outlet side of the flume, delineating a 9 m section as the fish breeding experimental zone.

A monitoring system, comprising nine infrared high-definition cameras (DS-2CD3T67WDV3-L, Hikvision Corporation, Hangzhou, China), a video recorder, and a monitor, was installed at the center of the flume to ensure continuous 24-h video recording. The video footage from all nine cameras was seamlessly integrated using Surfer 12 software, enabling efficient observation and documentation of fish reproductive activities. Human interference was minimized throughout the experiment to avoid disturbance to the silver carp. Additionally, the silver carp’s behavioral repertoire was expanded and refined through direct observations within the experimental area and visits to other original breeding farms of the FMCCs.

### 2.3. Experimental Methods

#### 2.3.1. Behavior Observation Experiment

During the silver carp breeding seasons of May and June in both 2023 and 2024, three spawning experiments were conducted each year. Each experiment involved the selection of three sexually mature females and four males. The male fish had an average body length of 76.47 ± 2.39 cm and an average weight of 7.91 ± 0.41 kg, while the female fish had an average body length of 79.11 ± 2.16 cm and an average weight of 9.34 ± 0.32 kg. The selected silver carp were administered an injection of compound chorionic gonadotropin type A (Ningbo Second Hormone Factory, Ningbo, China) at a dosage of 400 units/kg, with half the dosage for male fish. The injection was administered at the scaleless depression at the base of the pectoral fin around 10:00 am. After oxytocin injection, the flume’s impellers were promptly activated to stimulate estrus and spawning, with a water flow velocity of 0.4 to 0.5 m/s. To ensure optimal fish welfare and reproductive success, no anesthetic was administered prior to the oxytocin injection. Throughout the experiment, water temperature was maintained at 23.0 ± 1.0 °C, and the dissolved oxygen level was controlled at 8.5 ± 1.0 mg/L.

Preliminary findings indicate that the reproductive behaviors of silver carp predominantly occur within 20 h post-induction. Consequently, this study employed both focal animal sampling and instantaneous scan sampling methods to monitor silver carp behavior within the 0–20 h post-induction window [28]. Video recordings were utilized to develop an ethogram and document the occurrence of various behaviors. Concurrent manual observations supplemented the ethogram. Focal animal sampling focused on selecting silver carp displaying active behaviors and clear video footage as target subjects. Continuous 15-min observations were conducted to record all postures, movements, and environmental contexts of behaviors exhibited by these target individuals. After a 30-min interval, additional individuals meeting the same criteria were selected for further 15-min observations. Instantaneous scan sampling recorded the behaviors of all visible individuals within a few seconds at preset sampling time points, primarily to capture the frequency of each behavior. Each scan lasted approximately 1 min, with sampling repeated every 10 min to maintain relative independence of samples. The frequency of occurrence was calculated as the ratio of each reproductive behavior’s occurrence count to the total count of all observed reproductive behaviors. During quantitative video analysis, the footage was initially reviewed at 3 × speed using Smart Player (Version 3.41.0) software. Upon detecting target silver carp or reproductive behaviors, playback was switched to normal speed (1×) for precise behavioral frequency recording. Daytime was defined as the period from 6:00 a.m. to 7:00 p.m., based on time records from the automatic activation and deactivation of the camera’s supplementary lighting, while the remaining hours were classified as nighttime.

#### 2.3.2. Definition and Coding of Behavior

This study adopted the PAE coding method and the behavioral nomenclature framework for aquatic animals, as established by previous scholars [14,15,29], to categorize all recorded behaviors of silver carp during the breeding season into three hierarchical levels: posture (P code), action (A code), and environment (E code). “Posture” denotes the sustained state and spatial configuration of the primary structural components of silver carp over a defined duration. “Action” describes the movement of specific body parts, driven by muscle contraction, relaxation, displacement, and flexion within a short timeframe. “Environment” encompasses the specific conditions under which behavioral activities occur, classified into biotic and abiotic factors. Fish behavior emerges as a composite of postures and actions, modulated by environmental stimuli and exhibiting distinct adaptive mechanisms. By integrating the environmental context of each behavior, the sustained postures, and the executed actions, a PAE behavior coding system for silver carp during the breeding period was developed.

#### 2.3.3. Classification and Temporal Analysis of Reproductive Behaviors

**Figure 2:**
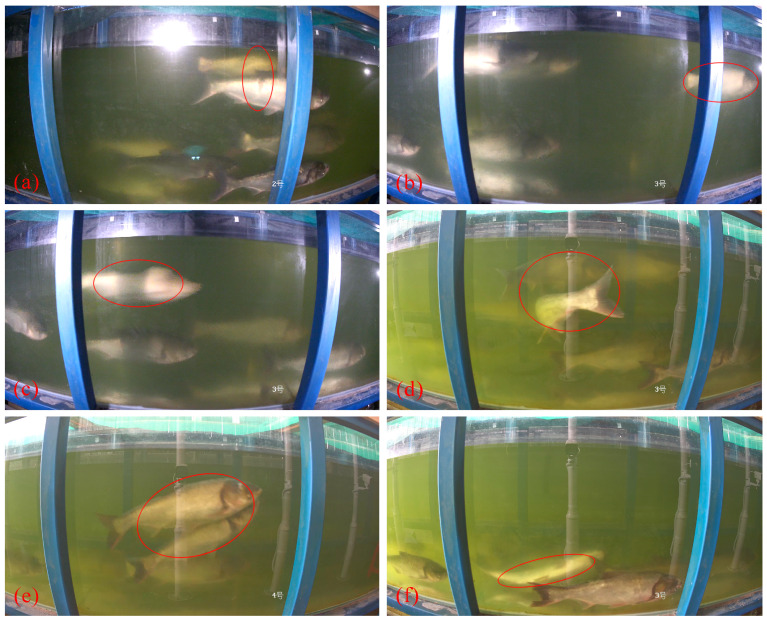
Partial reproductive behaviors of *Hypophthalmichthys molitrix*, as indicated by red circles: (**a**) The male and female fish swim upstream together; (**b**) A male fish leads the entire school of fish at the forefront; (**c**) A male fish accelerates towards nearby females; (**d**) The male and female fish bend their bodies towards each other, circling in place; (**e**) The male dives beneath the female and gently bumps her abdomen with his body; (**f**) The female lies on her side in the water, swaying her body while releasing her eggs.

Reproductive behaviors were categorized into six specific types: accompanying, guiding, chasing, encircling, tail-diving, and mating. Based on previous studies, the following definitions were applied to each behavior:

(1) Accompanying behavior: Males and females swim together slowly underwater, either upstream or downstream (Figure 2a).
(2) Guiding behavior: Within a moving school, a single male frequently assumes the leading position at the forefront (Figure 2b).
(3) Chasing behavior: Mature males accelerate toward nearby females, occasionally making physical contact with the female’s body (Figure 2c).
(4) Encircling behavior: Males and females engage in head-to-head contact, bending their bodies and circling in place by swaying their tails (Figure 2d).
(5) Tail-diving behavior: The male dives downward, approaches the female’s abdomen (Video S1), and gently bumps her genital opening with his head (Figure 2e).
(6) Mating behavior: The male presses against the female’s back, sometimes causing her to lie on her side in the water. Both fish contract and sway their bodies rhythmically, culminating in spawning (Videos S2 and S3) and ejaculation (Figure 2f).

Following the standardization of the classification of reproductive behaviors, the temporal sequence of reproductive behaviors in each silver carp was recorded through video observation to analyze the occurrence patterns of reproductive behaviors over time.

#### 2.3.4. Analysis of Reproductive Behavior Diversity

Drawing upon methodologies employed in behavioral diversity research on wild species such as leopard cats (*Prionailurus bengalensis*) [30] and forest musk deer (*Moschus berezovskii*) [31], the reproductive behavioral diversity index of silver carp was quantified. The absolute behavioral diversity index (*H*), maximum behavioral diversity index (*H*_max_), and behavioral diversity index for specific behavior types (*H*_variable_) were calculated based on different post-induction time intervals and sex-specific variations. Subsequently, the relative behavioral diversity index (*r*) and regulated diversity index (*r-variable*) were derived from these computations [32]. The calculation formulas for each index are presented as follows:

(1) $$H=–\sum_{i=1}^{s}(P_{i}\log_{2}P_{i})$$

(2) $$P_{i}=f_{i}/\sum f$$

In these formulas, *P_i_* represents the occurrence frequency of the *i*-th reproductive behavior of silver carp, and *f_i_* represents the occurrence count of the *i*-th reproductive behavior of silver carp.

(3) $$H_{\max}=\log_{2}N$$

In this formula, *N* represents the number of categories of reproductive behavior encompassed in the ethogram of silver carp.

(4) $$r=H/H_{\max}$$

(5) $$r-variable=H/H_{\;\mathrm{variable}}$$

(6) $$H_{\;\mathrm{variable}}=\log_{2}n$$

In these formulas, *n* represents the number of reproductive behavior elements specific to a particular time or group of silver carp.

### 2.4. Data Processing

The data collected in this study were categorized into qualitative and quantitative components. Qualitative data were employed to establish the PAE behavioral coding system, which systematically describes and classifies observed behaviors. Quantitative data were analyzed to compare differences in behavioral diversity indices and occurrence frequencies of reproductive behaviors across different post-induction intervals and between sexes in fish. Group differences were analyzed using one-way analysis of variance (ANOVA), followed by the least significant difference (LSD) test for multiple comparisons. All experimental results were expressed as mean ± standard deviation (SD), with statistical significance set at *p* < 0.05. Statistical analyses were performed using IBM SPSS Statistics 26, and graphical representations were generated with GraphPad Prism 9.

## 3. Results

### 3.1. Posture Coding

Through repeated observations and systematic identification, 12 distinct postures were recorded during the breeding period of silver carp: swimming, rushing, gliding, suspension, floating, adhering, jumping, ovipositing, turning, rotating, inverting, and sinking (Table 1). These postures were exhibited by both sexes. Among them, suspension, floating, and adhering were classified as static postures, while swimming, rushing, gliding, jumping, ovipositing, turning, rotating, inverting, and sinking were categorized as dynamic postures.

### 3.2. Action Coding

Based on the specific body parts engaged in silver carp movements, 20 distinct actions were identified and systematically coded (Table 2). The analysis revealed eight types of head-related actions, five of which involved the mouth: butting, spitting, gaping, swallowing, and breathing. Additionally, six body-related actions were recorded, while pectoral fin and caudal fin movements were the least frequent, each comprising three distinct action types.

### 3.3. Environmental Coding

Based on four key environmental factors—activity location, light conditions, sex, and shoaling pattern—the environmental contexts influencing silver carp behavior were categorized into 10 distinct types and systematically coded (Table 3). Among these, six were classified as abiotic environments: water surface, upper flume layer, lower flume layer, daytime, nighttime, and illuminated nighttime. The remaining four were categorized as biotic environments: male, female, solitary, and group.

### 3.4. PAE Coding System and Reproductive Ethogram

This study documented 34 behavioral patterns exhibited by silver carp during the breeding period. Based on their biological functions, these behaviors were classified into five major categories: feeding and excretion, locomotion, aggregation, reproduction, and miscellaneous behaviors. The PAE element coding system for silver carp during the breeding period was developed by systematically integrating posture codes, action codes, and environmental codes (Table 4).

During the experimental period, the frequency of each behavioral type observed among the 42 silver carp individuals is presented in Table 4. All six reproductive behaviors were exhibited in the majority of the test subjects. Temporal analysis of reproductive behaviors across the 42 specimens revealed chronobiological disruption, characterized by two primary abnormalities: First, the courtship-spawning behavioral sequence exhibited partial fragmentation, with 64.29% of specimens proceeding directly to oviposition without completing characteristic courtship rituals (e.g., encircling and tail-diving displays). Second, synchronization of gamete release was markedly impaired, with only 30.56% of instances demonstrating temporal coordination between sperm ejection and ovulation.

### 3.5. Reproductive Behavior Diversity of Silver Carp

#### 3.5.1. Differences Among Groups with Varying Durations Following Induced Spawning

**Figure 3:**
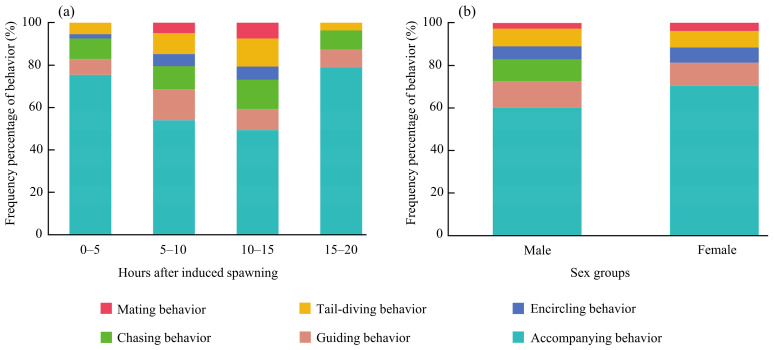
The frequency percentage of reproductive behavior in *Hypophthalmichthys molitrix* across different time intervals after induced spawning (**a**) and between sexes (**b**).

This study demonstrated that the reproductive behaviors of silver carp varied across different post-induction time intervals. All reproductive behaviors were observed within the 5–10 h and 10–15 h intervals following induced spawning (Figure 3a). In contrast, only five and four reproductive behaviors were recorded during the 0–5 h and 15–20 h intervals, respectively. Notably, mating behavior was absent in both the 0–5 h and 15–20 h intervals, and encircling behavior was not observed during the 15–20 h interval (Figure 3a). Among all reproductive behaviors, accompanying behavior exhibited the highest frequency percentage across different time intervals. Guiding behavior was more frequently observed during the 5–10 h interval than at any other time. The frequency percentages of chasing, encircling, tail-diving, and mating behaviors were highest within the 10–15 h interval, exceeding those recorded during other time periods.

Regarding behavioral diversity indices, *H*, *r*, and *r-variable* values peaked during the 10–15 h interval and reached their lowest levels in the 15–20 h interval (Table 5). As time progressed post-induction, these indices initially increased before declining. Significant differences were observed among different time interval groups (*p* < 0.001). Pairwise comparisons indicated that the difference in *r-variable* values between the 0–5 h and 15–20 h groups was not significant (*p* > 0.05), while all other pairwise comparisons showed statistically significant differences (*p* < 0.001).

#### 3.5.2. Gender Differences

Male silver carp exhibited all six reproductive behaviors during the breeding period, while females engaged in only five, with the absence of chasing behavior. Among both sexes, accompanying behavior occurred most frequently. Females showed a higher frequency of accompanying, encircling, and mating behaviors than males, while other reproductive behaviors were less common in females (Figure 3b). All diversity indices—*H*, *r*, and *r-variable*—were substantially higher in males, with differences in all measures being highly significant (*p* < 0.001).

## 4. Discussion

### 4.1. The Ethogram of Silver Carp During the Breeding Period

This study replicated the natural habitat of silver carp in a large indoor flume, continuously monitoring their behavior across two breeding seasons and identifying five categories encompassing 34 distinct behaviors. Compared to other aquatic animals, silver carp exhibited a relatively constrained behavioral repertoire. For instance, *Schizothorax wangchiachii* in the upper Yangtze River displayed 43 behavioral patterns in natural water bodies [15], while 61 were documented for Yangtze finless porpoises in the Nanjing section of the Yangtze River [14]. This disparity likely arises from the larger habitat space and more diverse social interactions found in natural environments. Under the controlled indoor conditions of this study, silver carp exhibited breeding behaviors within a limited spatial range and an environment of reduced complexity, resulting in behavioral distinctions from wild aquatic animals. Consistent with these findings, Xiang et al. [16] identified 29 breeding-related behaviors in *Odontobutis potamophila* through a similar indoor pond simulation. Moreover, the ethogram of PAE behaviors in aquatic animals, particularly fish, is generally more rudimentary than that of higher terrestrial animals. Previous studies have recorded up to 134 behavioral types in Père David’s deer [9] and 163 in the Guizhou snub-nosed monkey (*Rhinopithecus brelichi*) [33], highlighting a significant contrast in behavioral complexity between aquatic and terrestrial species. The disparity in behavioral complexity primarily stems from differences in evolutionary development and habitat characteristics between terrestrial and aquatic animals [34]. Over evolutionary timescales, many terrestrial species have developed more advanced nervous systems and locomotor structures, facilitating a broader and more intricate range of behaviors. In contrast, fish and other aquatic animals generally occupy a lower evolutionary tier [35], characterized by relatively primitive cranial musculature and limited muscle diversity, which constrain their postural variations, movement patterns, and behavioral complexity. As a result, their capacity for facial expressions is also markedly restricted [36]. Furthermore, terrestrial ecosystems present a greater diversity of environmental challenges, including varied terrain, abundant shelter, and a higher density of predators. These factors necessitate adaptive behavioral strategies that align with the physiological traits and ecological pressures of terrestrial species, fostering the evolution of more complex and dynamic behavioral repertoires [37]. In contrast, aquatic environments remain comparatively stable, imposing fewer selective pressures that drive behavioral diversification. In this study, silver carp primarily inhabited the upper and middle layers of the water column, where environmental homogeneity and limited social interactions further constrained behavioral complexity, resulting in a relatively simple ethogram.

### 4.2. The Reproductive Behavior Characteristics of Silver Carp

Reproduction represents a pivotal phase in the life history of fish, during which distinct behavioral patterns emerge at different stages. This study demonstrated that all categories of reproductive behaviors in silver carp occurred within the 5–10 h and 10–15 h intervals following induced spawning, whereas mating behavior remained absent during both the initial 0–5 h and subsequent 15–20 h periods. Previous research has established that water flow and artificial induction can effectively stimulate gonadotropin secretion in bony fish, thereby enhancing reproductive behaviors in broodstock and facilitating successful spawning [38,39]. However, the hormonal regulation of germ cell maturation and mating behavior is time-dependent, exerting prolonged effects that synchronize sexual maturation and ovulation [40,41]. Consequently, estrous and mating behaviors are confined to specific temporal windows, a phenomenon similarly observed in avian reproductive patterns by Hoekzema et al. [42]. Future research should explore the impact of varying flow rates and oxytocin dosages on the natural reproduction of silver carp, providing a scientific basis for artificial breeding strategies, reproductive behavior analysis, and wild population conservation. In addition, the reproductive behavior diversity index of silver carp peaked at 10 and 15 h post-induction, with significantly lower values during the 0–5 h and 15–20 h periods. These results indicate that the most intensive mating activities occur between 10 and 15 h post-spawning, coinciding with the highest behavioral diversity. This temporal pattern may be influenced by environmental factors regulating species-specific behaviors [43]. The confined flume environment may alter behavioral structures, potentially suppressing certain actions, such as guiding behavior, thereby contributing to the reduced behavioral diversity index observed during the initial 0–5 h period [44]. Conversely, oxytocin stimulation and water flow enhancement significantly intensified reproductive activity, leading to a marked increase in behavioral diversity between 10 and 15 h post-induction. Once mating behaviors concluded, silver carp entered an energy recovery phase characterized by accompanying swimming, which corresponded with the decline in behavioral diversity during the 15–20 h period.

Beyond temporal variations in reproductive behaviors following induced spawning, gender differences also significantly influence behavioral diversity in silver carp. In this study, females exhibited lower diversity and intensity in reproductive behaviors compared to males throughout the breeding process. Females predominantly remained in a stable accompanying state, with no engagement in chasing behavior, whereas males displayed greater activity and initiative, frequently performing guiding, chasing, and tail-diving behaviors. These findings align with behavioral observations in *Schizothorax wangchiachii* [15]. The observed gender differences likely reflect distinct reproductive strategies. Females allocate the majority of their time and energy to oocyte development, while males focus on securing mating opportunities through competitive and courtship behaviors [45]. This strategy allows reproductively dominant males to maximize their mating success and, consequently, enhance the survival potential of their offspring [46,47].

Previous studies suggest that migratory fish species (e.g., sturgeons) rely on specific hydraulic conditions, particularly flow velocities exceeding 1.0 m/s, to trigger gonad development and facilitate spawning over suitable riverbed substrates [48,49]. This spawning sequence follows a distinct temporal pattern: substrate exploration precedes gamete release, optimizing egg survival by ensuring high-oxygen microhabitats and reducing predation pressure [50]. However, this study revealed significant temporal disruption in the reproductive behaviors of silver carp, potentially attributable to two key factors. First, the experimental tank environment lacked the eco-hydraulic conditions characteristic of natural spawning grounds (e.g., specific flow regimes and substrate textures). Second, the broodstock’s courtship behaviors may have degenerated after multiple generations of artificial cultivation in fish farms [51,52]. Furthermore, due to the prolonged absence of multi-modal environmental cues (e.g., hydrological pulses and chemical signals) present in natural river systems, the fish exhibited aimless swimming under experimental conditions, which likely contributed to asynchronous gamete release [53]. It is noteworthy that the reduced synchrony of sperm-egg release inevitably decreases fertilization rates [54], consequently leading to a decline in natural population recruitment of silver carp. This phenomenon is particularly pronounced in the middle reaches of the Yangtze River, where the construction and operation of the Three Gorges Project have significantly altered the geomorphological and hydrodynamic conditions of traditional spawning grounds for the FMCCs [55,56]. Specifically, the decreased flow velocity during spawning seasons has impaired gamete encounter efficiency, resulting in persistent shrinkage of spawning ground areas [57]. These findings further elucidate the cascade mechanism of “hydrological alteration → behavioral disruption → reproductive failure → population decline”, providing critical evidence for river basin ecological restoration.

### 4.3. The Significance of the PAE Coding System in the Study of Animal Behavior

The PAE coding system for animal behavior aims to standardize the dimensions of behavioral analysis and streamline the recording process [58]. However, current research on animal ethograms is largely qualitative, with varying descriptive definitions of identical behaviors across different scholars [59], lacking a systematic approach and standardized framework for behavior classification. In recent years, the PAE coding system has emerged as an innovative methodology that integrates posture, action, and corresponding environmental elements, becoming widely used for the classification and quantification of animal behaviors. For example, chasing behavior in silver carp is observed in the upper layer of the flume under different lighting conditions, manifesting as a shoaling pattern where males pursue females (environmental codes: 2, 4, 5, 6, 7, 8, 10). Males primarily exhibit postures such as swimming, rushing, turning, and rotating (posture codes: 1, 2, 9, 10), frequently raising and shaking their heads, with their bodies extending, bending, and turning left and right. Additionally, actions such as stretching their pectoral fins and swinging their caudal fins continuously are displayed (action codes: 6, 8, 9, 10, 13, 14, 15, 19). This study on behavioral patterns has not only enhanced the standardization and reliability of fish behavior ethograms but also facilitated a deeper understanding of the relationship between animal behavior and its biological functions [60,61]. Moreover, it provides a key foundation for uncovering the intrinsic connections between behavior and biological processes.

Furthermore, the PAE coding system significantly enhances our understanding of the relationship between animal behavior and environmental adaptability. For instance, Luo et al. [62] demonstrated that, by using the PAE coding system to quantify the behavioral characteristics and environmental thresholds of the Chinese giant salamander (*Andrias davidianus*) during its breeding period, it becomes possible to reveal the behavioral ecological patterns and the habitat requirements essential for its reproduction. Additionally, applying PAE coding to animal behavior offers valuable insights for assessing the adaptability of endangered species to environmental changes and evaluating the effectiveness of rewilding efforts. This approach provides a scientific foundation for the conservation and management of endangered species [14,63,64]. Behavioral diversity reflects a species’ ability to adapt to its environment. Research by Liu et al. [65] and Zhou et al. [66] conducted quantitative analyses of animal behavioral diversity using the PAE ethogram, uncovering differences in behavioral patterns across various wildlife species as they adapt to different environmental conditions. This offers new perspectives for studying animal behavior. In summary, the PAE coding system elevates the scientific rigor and precision of animal behavior research through standardized, accurate, and structured behavioral recording. Its extensive potential applications include the exploration of behavioral patterns, cross-species comparisons, conservation of endangered species, and the quantitative analysis of behavioral diversity.

### 4.4. Management Implications

Animal behavioral plasticity, as an adaptive phenotypic response to environmental heterogeneity, can effectively characterize species-specific physiological adaptation strategies and ecological requirements [37,67]. This study, through controlled laboratory experiments, has for the first time established an ethogram for silver carp during its reproductive period and quantitatively characterized its behavioral patterns and diversity features. The findings not only reveal the functional relationships between silver carp’s reproductive behaviors and hydrological environmental factors but also provide a scientific basis for optimizing artificial breeding protocols and wild population conservation strategies. Based on the research findings, the following management recommendations are proposed:

(1) Optimization of artificial breeding techniques. Priority should be given to selecting broodstock exhibiting high behavioral synchrony (e.g., complete courtship rituals) in order to enhance artificial reproduction quality. Intermittent water flow (alternating between 0.5 and 1.5 m/s) should be introduced into the aquaculture system to restore natural reproductive behavioral competence in captive populations. Additionally, wild individuals should be periodically integrated into the breeding stock to mitigate the potential degradation of reproductive behaviors that may result from artificial selection.
(2) Strategies for the conservation of wild populations. In conjunction with the ecological scheduling of the Three Gorges Reservoir, the natural hydrological flood peak process should be simulated from April to June to stimulate the natural reproductive behavior of fish. Efforts should be intensified to restore the spawning grounds of the FMCCs and protect their habitats. Furthermore, the synchronization rate of sperm and egg release should be incorporated into the health assessment framework for Yangtze River spawning grounds as a quantitative indicator of reproductive success.

## 5. Conclusions

This study documented the behavioral patterns of silver carp during the breeding period through video monitoring, manual observation, and field investigations. Notably, it established the first behavioral ethogram for the species and developed a corresponding PAE coding system, identifying 12 postures, 20 actions, and 34 distinct behaviors. Quantitative analysis enabled the calculation of reproductive behavior frequency and diversity across different time intervals following induced spawning, as well as between sexes. These findings fill a critical gap in the behavioral ethogram of silver carp, providing a robust foundation for systematically exploring the ecological mechanisms underlying their behavior. Moreover, this study serves as a valuable reference for constructing behavioral ethograms and analyzing behavioral diversity in fish and other aquatic species. Given the challenges of real-time behavioral observation in wild silver carp populations, reproduction experiments were conducted in a large indoor circular flume, differing from natural spawning habitats. Future research should incorporate underwater video monitoring in natural spawning grounds to capture more comprehensive behavioral data from breeding individuals, refining the behavioral ethogram and enhancing the accuracy of reproductive behavior characterization in silver carp.

## Figures and Tables

**Table 1:** Posture codes for Hypophthalmichthys molitrix.

Posture	Definition	Code
Swimming	The silver carp swims freely in the water by swinging its pectoral and caudal fins.	1
Rushing	The caudal fin of the silver carp rapidly undulates, enabling it to swim swiftly through the water.	2
Gliding	The silver carp maintains a fixed posture as it slows down and glides through the water.	3
Suspension	The body remains suspended in the water, with the pectoral fin moving slightly or remaining stationary.	4
Floating	The body floats at the water surface, with the pectoral fin moving slightly or remaining stationary.	5
Adhering	The silver carp adheres to the bottom of the water tank, with its body remaining motionless.	6
Jumping	The tail fin suddenly exerts force, propelling the body upwards.	7
Ovipositing	The female fish flips its body, and as its pectoral and pelvic fins quiver, it begins to spawn.	8
Turning	The trunk of the silver carp moves laterally along the dorsoventral axis.	9
Rotating	The trunk of the silver carp rotates around the longitudinal axis.	10
Inverting	The trunk of the silver carp is inverted along the dorsoventral axis, with the abdomen on top and the back on the bottom.	11
Sinking	The silver carp remains motionless and falls freely towards the bottom of the water tank.	12

**Table 2:** Action codes for *Hypophthalmichthys molitrix*.

Body Position	Action	Code
Head	Butting	1
	Spitting	2
	Gaping	3
	Swallowing	4
	Breathing	5
	Heading up	6
	Heading down	7
	Shaking	8
Trunk	Extending	9
	Bending	10
	Swinging	11
	Rubbing	12
	Turning left	13
	Turning right	14
Pectoral fin	Stretching	15
	Swinging	16
	Retracting	17
Caudal fin	Extending	18
	Continuous swinging	19
	Intermittent swinging	20

**Table 3:** Environment codes for *Hypophthalmichthys molitrix*.

Classification	Environment	Biotic (E1)	Abiotic (E2)	Codes
Activity location	Water surface		+	1
	Upper flume layer		+	2
	Lower flume layer		+	3
Light conditions	Daytime		+	4
	Nighttime		+	5
	Illuminated nighttime		+	6
Sex	Male	+		7
	Female	+		8
Shoaling pattern	Solitary	+		9
	Group	+		10

Note: The symbol “+” indicates that it belongs to this type.

**Table 4:** PAE coding system for *Hypophthalmichthys molitrix* during the breeding period.

Behavior	Behavioral Performance Quantity	Male	Female	Number	PAE Code		
					P	A	E
**Feeding and excretion** **behavior**							
Swallowing	40/42	++	++	1	1, 4, 6	3, 4, 5, 6, 9, 17, 20	2, 3, 4, 5, 6, 7, 8, 9
Filtering	42/42	+++	++	2	1, 4, 6, 9	3, 4, 9, 13, 14, 17, 18, 20	2, 3, 4, 5, 6, 7, 8, 9, 10
Spiting	42/42	++	++	3	1, 3, 4, 6	3, 7, 9, 13, 14, 17, 20	2, 3, 4, 5, 6, 7, 8, 9
Defecating	42/42	+	+	4	1, 3, 4, 6, 9	6, 9, 13, 14, 17, 20	3, 4, 5, 6, 7, 8, 9
**Locomotion behavior**							
Observing	42/42	++	++	5	1, 3, 6, 12	6, 7, 8, 9, 13, 14, 17, 18, 20	2, 3, 4, 6, 7, 8, 9, 10
Patrolling	41/42	++	+	6	1, 9	8, 9, 13, 14, 16, 17, 20	2, 4, 6, 7, 8, 9
Fast-start	32/42	++	+	7	1, 2, 3, 9	8, 9, 10, 17, 19	2, 3, 4, 7, 8, 9
Vertical jumping	18/42	+		8	2, 7, 9	6, 9, 10, 15, 17, 19	1, 4, 5, 6, 7, 8, 9
Goring jumping	29/42	++	+	9	2, 7, 9	6, 9, 10, 15, 17, 19	1, 4, 5, 6, 7, 8, 9
Sweeping water	32/42	++	++	10	1, 2, 7, 9	6, 8, 9, 10, 11, 15, 19	1, 2, 4, 6, 7, 9
Diving	30/42	+	++	11	1, 12	5, 7, 9, 17, 18, 20	3, 4, 5, 6, 7, 8, 9
Floating	36/42	++	++	12	1, 5	5, 8, 9, 16, 17, 20	1, 4, 5, 6, 7, 8, 9
Swimming	42/42	+++	+++	13	1, 2, 7, 9	8, 9, 10, 13, 14, 16, 17, 19, 20	E1, E2
Slowly swimming	42/42	+++	+++	14	1, 9	5, 8, 9, 13, 14, 17, 18, 20	E1, E2
Inverse swimming	42/42	+++	+++	15	1, 2, 7, 9	8, 9, 10, 13, 14, 16, 17, 19	2, 3, 4, 5, 6, 7, 8, 9, 10
Swimming downstream	14/42	+		16	1, 3, 9, 10	8, 9, 13, 14, 17, 18	1, 2, 4, 5, 6, 7, 8, 9
Detecting object	39/42	+	++	17	1, 2, 3, 6, 9	1, 7, 8, 9, 12, 13, 14, 17, 20	2, 3, 4, 5, 6, 7, 8, 9
**Aggregation behavior**							
Group touring	42/42	+++	+++	18	1, 2, 9	8, 9, 13, 14, 17, 20	2, 3, 4, 6, 7, 8, 10
Searching	42/42	+++	++	19	1, 2, 9, 10	6, 7, 8, 9, 13, 14, 17, 20	2, 3, 4, 6, 7, 8, 10
Following	42/42	+++	+++	20	1, 9, 10	6, 7, 8, 9, 10, 13, 14, 17, 20	2, 3, 4, 6, 7, 8, 10
Clustering	42/42	+++	++	21	1, 4, 6	6, 7, 8, 9, 13, 14, 16, 17, 18, 20	2, 3, 4, 5, 6, 7, 8, 10
Disperse	40/42	++	+	22	1, 2, 9, 10	6, 7, 8, 9, 10, 13, 14, 16, 19, 20	2, 3, 4, 5, 6, 7, 8, 10
Avoiding	39/42	++	+	23	1, 9, 10	8, 9, 13, 14, 15, 16, 19, 20	2, 3, 4, 5, 6, 7, 8, 10
Leaving	37/42	++	+	24	1, 9	8, 9, 13, 14, 15, 16, 20	2, 3, 4, 5, 6, 7, 8, 10
Frolic	35/42	+	++	25	1, 2, 9	1, 8, 9, 11, 13, 14, 17, 18, 20	1, 2, 4, 6, 7, 8, 10
**Reproduction behavior**							
Accompanying	42/42	+++	+++	26	1, 9, 10	8, 9, 10, 13, 14, 17, 18, 20	2, 3, 4, 5, 6, 7, 8, 10
Guiding	37/42	++	+	27	1, 9, 10	8, 9, 10, 13, 14, 17, 18, 20	2, 3, 4, 5, 6, 7, 8, 10
Chasing	23/42	+		28	1, 2, 9, 10	6, 8, 9, 10, 13, 14, 15, 19	2, 4, 5, 6, 7, 8, 10
Encircle	28/42	+	+	29	1, 5, 9, 10	1, 8, 10, 12, 13, 14, 16, 19	1, 2, 4, 5, 6, 7, 8, 10
Tail-diving	31/42	+	+	30	1, 2, 9, 12	1, 6, 7, 9, 12, 15, 16, 20	3, 4, 5, 6, 7, 8, 10
Mating	37/42	+	+	31	1, 4, 8, 9, 10, 11, 12	1, 6, 9, 10, 11, 12, 16, 18, 20	2, 3, 4, 5, 6, 7, 8, 10
**Miscellaneous behavior**							
Breathing	42/42	+++	+++	32	1–12	5	E1, E2
Sound production	16/42	+	+	33	7	3, 10, 11, 17, 19	1, 4, 7, 9
Splitting	34/42	++	++	34	1, 4, 5, 6, 11	2, 3, 9, 17, 20	1, 2, 3, 5, 7, 9

Note: “+” denotes the likelihood of a behavior occurring, with an increasing number of “+” symbols indicating a higher frequency of occurrence.

**Table 5:** The diversity index of reproductive behavior of *Hypophthalmichthys molitrix* among different groups.

Groups	Classification	Absolute Behavioral Diversity Index (*H*)	Relative Behavioral Diversity Index (*r*)	Regulated Diversity Index (*r-Variable*)
Groups with varying durations following induced spawning	0–5 h	1.25 ± 0.05 ^c^	0.49 ± 0.02 ^c^	0.54 ± 0.02 ^c^
	5–10 h	2.01 ± 0.04 ^b^	0.78 ± 0.02 ^b^	0.78 ± 0.02 ^b^
	10–15 h	2.14 s 0.01 ^a^	0.83 ± 0.00 ^a^	0.83 ± 0.00 ^a^
	15–20 h	1.05 ± 0.04 ^d^	0.41 ± 0.02 ^d^	0.52 ± 0.02 ^c^
*p*-value		*p* < 0.001	*p* < 0.001	*p* < 0.001
Gender groups	Male	1.84 ± 0.04 ^a^	0.71 ± 0.01 ^a^	0.71 ± 0.01 ^a^
	Female	1.44 ± 0.03 ^b^	0.56 ± 0.01 ^b^	0.62 ± 0.01^b^
*p*-value		*p* < 0.001	*p* < 0.001	*p* < 0.001

Note: Values in the same column not sharing the same superscript letter are significantly different (*p* < 0.001).

## Data Availability

The data presented in this study are available on request from the corresponding author.

## Supplementary Materials

The following supporting information can be downloaded at: https://www.mdpi.com/article/10.3390/ani15091218/s1, Video S1: The tail-diving behavior involves the male diving beneath the female and gently bumping her abdomen with his body. Video S2: The process in which the female lies on her side in the water, swaying her body while releasing eggs. Video S3: Close-up video footage of the female spawning in an upright position.
