# Supplementary material for: Ethogram Characteristics of Silver Carp (Hypophthalmichthys molitrix) During the Breeding Period Based on the PAE Coding System
Source: Animals (Basel). 2025 Apr 25;15(9):1218. doi: 10.3390/ani15091218 (PMC12070832; doi:10.3390/ani15091218)
Supplement: Supplementary file 1 [file animals-15-01218-s001.zip › Supplementary Materials.pdf]

Supplementary Materials: Video S1: The tail-diving behavior involves the male diving beneath the female and gently bumping her abdomen with his body. Video S2: The process in which the female lies on her side in the water, swaying her body while releasing eggs. Video S3: Close-up video footage of the female spawning in an upright position.
